# Non-disease Specific Health Promotion Interventions for Chronically Ill Adolescents in Medical Settings: A Systematic Review

**DOI:** 10.3389/fpubh.2018.00301

**Published:** 2018-11-13

**Authors:** Isabelle Aujoulat, Sophie Dechêne, Magali Lahaye

**Affiliations:** ^1^Institute of Health and Society, Université Catholique de Louvain, Brussels, Belgium; ^2^MRCPsych, Department of Child Psychiatry, Institute of Health and Society, Université Catholique de Louvain, Cliniques Universitaires Saint-Luc, Brussels, Belgium; ^3^Department of Pediatric Hematology and Oncology, Institute of Health and Society, Université Catholique de Louvain, Cliniques Universitaires Saint-Luc, Brussels, Belgium

**Keywords:** adolescent, health promotion, chronic condition, hospital, skills for health

## Abstract

Adolescents with chronic conditions are highly likely to encounter physical, social and psychological difficulties that can threaten their overall wellbeing and health. As any other adolescent, they need to be helped to tackle the non-medical determinants of their health. This is the aim of primary prevention and general health promotion interventions. The present paper aims to review any hospital-based intervention that strives to promote general health in chronically ill teenagers. A systematic process of search and screening revealed four articles that presented and evaluated non-disease specific interventions that explicitly aimed to promote the overall health of chronically ill teenagers in clinical settings. Congruently with health promotion principles and values, the interventions described in our selection of articles targeted positive health determinants, in terms of personal skills and attitudes that contribute to psychosocial resiliency. The clinical relevance and feasibility of developing non-disease specific health promotion interventions in clinical settings was confirmed. However, the lack of relevant reported details did not allow us to highlight the key factors and mechanisms associated with successful interventions for health promotion targeted at chronically ill adolescents attending health care settings. Moreover, the design of the included studies varied in quality: number of participants, presence of a post-test and a follow-up, use of validated questionnaires, etc. Well-conducted non-disease specific clinical health promotion interventions still remain an under-investigated area of research, and maybe even of practice.

## Introduction

In industrialized countries, the number of adolescents suffering from a chronic medical condition has increased greatly in the last few years (1). In particular, the prevalence of common adolescent conditions, such as diabetes (2), asthma (3), obesity (4), or acne (5) is increasing. The percentage of adolescents suffering from a condition that lasts longer than 6 months is about 20–30% (6). The definition of a chronic medical condition is any physical condition that impacts daily activities and requires regular treatment and/or regular clinic attendance.

Living with a chronic illness and dealing with an ongoing treatment is a serious challenge, especially during adolescence. Indeed, this developmental stage is characterized by a process of individuation, through which teenagers build their own sense of identity, and may be accompanied by risk-taking behaviors (7, 8). Such behaviors can be hazardous in the short-term, by directly endangering a teenager's health or life or, in the longer term, by affecting their general physical, psychological or social health (9). The journey through adolescence for those with a chronic illness is significantly more challenging and there is some evidence that these youth may be more likely to internalize their problems (10) and suffer from depressive symptoms (11). They may also be more inclined to engage in health damaging behaviors such as smoking, unsafe sexual practices, and the use of alcohol or illicit drugs (12–14). Moreover, in adolescents with chronic conditions, risk-taking behaviors may specifically translate into non-adherence to prescribed treatments. Such behaviors can have a deleterious effect on any chronic condition and increase these adolescents' vulnerability to severe health consequences, in the short or long term. Nevertheless, such behaviors are to be considered as inherent to adolescence and part of the process of growth and individuation (15, 16).

The process of identity formation is likely to be challenged in chronically ill adolescents (17). As a consequence of the numerous physical, psychosocial or cognitive changes they have to deal with (18), as well as the limitations in their activities, they may be more likely to experience difficulties in peer-relationships (19). Overall their physical and psychosocial development influences their chronic condition as much as their chronic condition influences their bio-psychosocial growth. A multidisciplinary approach to provide chronically ill adolescents with support and guidance is therefore crucial to help them through puberty, and cope with their condition in a constructive way (20).

In the early 1990s, following the recommendation issued in the Ottawa Charter for Health Promotion (21), to “*Reorient Health Services*” to better meet the health needs of individuals as whole persons, the WHO launched the Health Promoting Hospitals network (HPH), acknowledging that hospitals have a role to play in order to promote people's health, alongside providing traditional medical services (22). At the 11th International Conference on Health Promoting Hospitals in 2003, child and adolescent patients were acknowledged as an important target group within the HPH network, as their health and developmental needs ought to be addressed jointly through empowering health promoting interventions. Following on from a literature review performed in 2003 that explored young people's health promotion needs in clinical settings (23), we aimed, with the present study, to identify practices that support health promotion needs in adolescents living with chronic conditions.

Health promotion interventions are defined as a set of interventions that “predisposes, enables and reinforces people to take greater control of the non-medical determinants of their own health” (24). These are driven by the aim to enhance individual empowerment. In chronically ill people, and more particularly in adolescents, the process of empowerment is not only about managing illness or treatment; it is also about developing self-determination and self-regulation competences while developing a valuable sense of self and meaningful social interactions with others (25, 26). Although illness education and self-management support interventions are of utmost importance to help young patients cope with the challenges related to their condition, in the context of this review, we focused on non-disease specific health promotion and primary prevention interventions that targeted adolescents with different chronic conditions. As highlighted by Sawyer et al. (27), research on adolescents with chronic conditions tends to focus on disease-specific challenges, with the risk of undermining a more generic understanding of common challenges and opportunities to improve the provision of care for young patients.

The overall aim of the present review is therefore to identify any non-disease specific intervention that promotes chronically ill adolescents' health in medical settings.

More specifically, our review aims to answer the two following research questions:

- What are the characteristics of the available non-disease specific health promotion interventions targeted at adolescents with chronic conditions in clinical settings?
- What are the targeted outcomes and how are the effects of these interventions assessed?

## Methods

### Search strategy

Information sources included four databases: Pubmed, Scopus, CINHAL, and Psychinfo. In each database, we conducted the literature search using combinations of the following keywords in titles and abstracts: (“ado^*^”) AND (“hospital” or “day care” or “ambulatory care” or “outpatient care” or “chronic disease” or “chronic condition” or “chronic illness” or “long-term disease” or “long-term condition” or “long-term illness”) AND (“health promotion” or “health education” or “prevention”). Only the word “ado^*^” (alternatively derived terms such as adolescent(s) or adolescence”) was included in the search as the terms “youth” and “teenager” did not yield different results.

Filters used to select the relevant articles were: only original manuscripts, articles including ages between 12 and 18, articles written in French, English, Italian, Portuguese or Spanish, articles from developed countries (as the scope of health promotion may be different in developing countries, where people find it more difficult to access fairly good quality and affordable medical care), and articles written from 2003 to 2017. The starting date (i.e., 2003) was determined on the basis that the needs and expectations of a generation of adolescents differ from one another, mentalities evolve and the patient/professional relationships tend to change as well. This date also correlates with the explosion of internet use among teenagers, which has greatly changed their connection to the world and access to knowledge (28). Moreover, another review investigating young people's health promotion needs in clinical settings was conducted in 2003 (23). An initial search was performed in 2014 for the 2003–2013 period, and an update was done in early 2018 to cover the 2014–2017 period.

Interventions were only included if they were non-disease specific, if they had been evaluated, and if they looked at the primary prevention of risk behaviors or at the promotion of general skills for health. Interventions specifically targeting secondary prevention (screening for medical disorders) and tertiary prevention (treatment of a diagnosed condition or an established risk behavior associated with a chronic condition) were excluded. For example, interventions aimed to reduce unhealthy eating habits in obese adolescents would be considered tertiary prevention and not be included in our review. We did not exclude interventions that targeted both children and teenagers, provided that results pertaining to the sub-group of teenagers were well reported.

Our initial search yielded 6,765 titles. Following the screening of titles and removal of duplicates, the abstracts of 3,246 articles were screened by independent reviewers. Based on the screening of abstracts, 174 full-text articles were retrieved for further consideration for inclusion. These 174 articles were read by two of the three reviewers (ML, SD, IA). Any disagreement was resolved through discussion between the three reviewers. This step led to the exclusion of a further 170 articles, based on the following exclusion criteria:

- studies focused on disease-specific interventions
- interventions took place in non-medical settings
- interventions targeted healthy adolescents rather than the chronically ill ones
- studies failed to give a detailed assessment of the intervention
- studies did not evaluate the effect of the intervention on the adolescents
- studies aimed at secondary or tertiary prevention (such as therapeutic patient education and self-management support) rather than primary prevention

**Figure 1:**
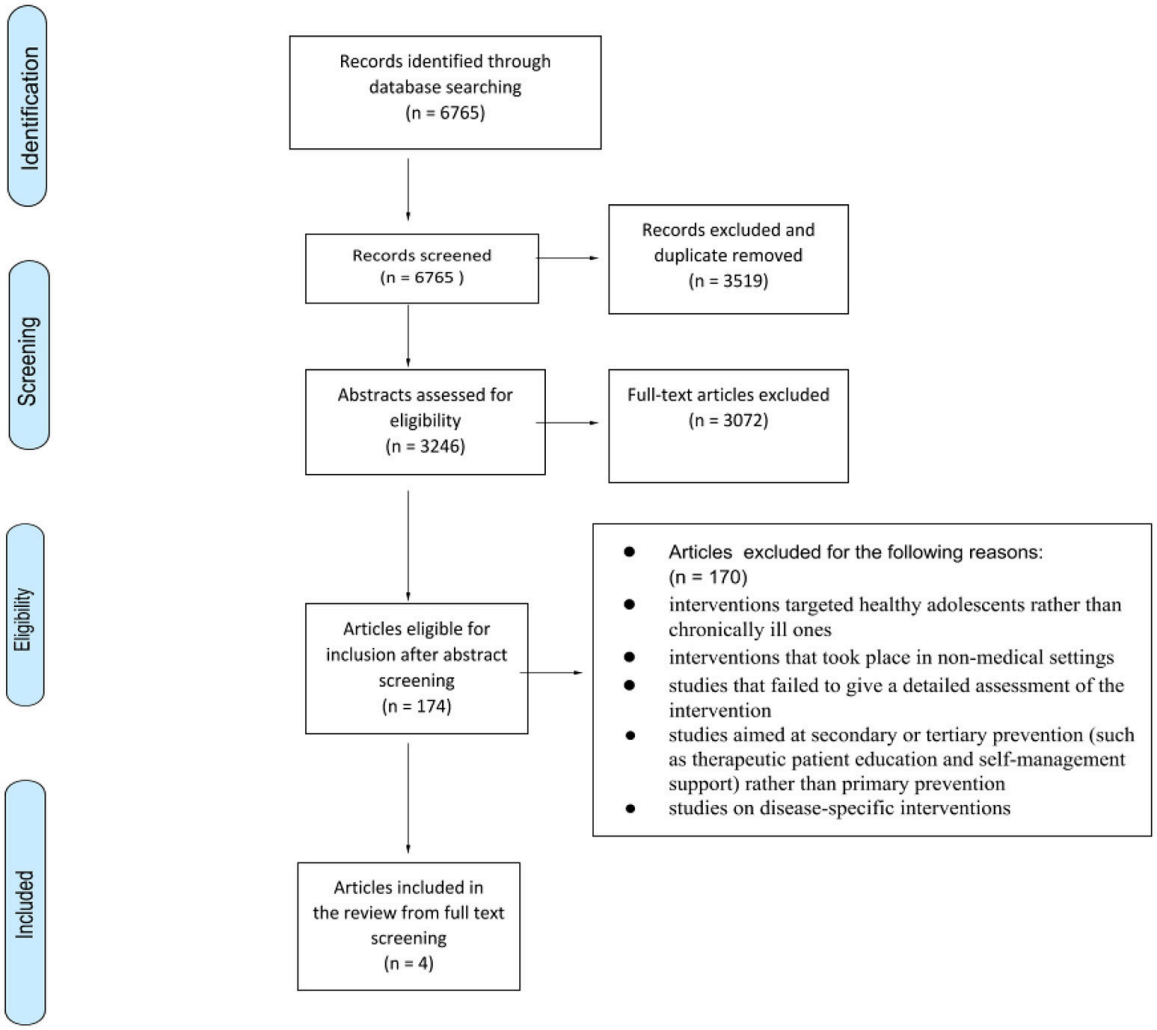
Flow diagram of selection process.

Our analysis hereafter is therefore based on a final selection of 4 studies. The study selection based on Prisma flow of information chart is detailed in Figure 1 (29).

### Data analysis

Data relating to conception, implementation and evaluation of the interventions were extracted from each study by two independent reviewers (ML and SD). In case of disagreement, a third author (IA) was consulted. Data relating to the conception of the interventions included information regarding the reasons behind the study (authors' motivation), the intervention's theoretical or ethical background and the role of the beneficiaries in designing the intervention (if involved).

Data from the implementation of the interventions included a description of the target population, context, aim, objectives, and methods. We also looked at the type of professionals involved at different stages of the interventions, and their respective roles.

Data from the evaluation of the interventions were extracted to document the effectiveness of the interventions. We looked at the objectives, methods and type of indicators used.

Moreover, to assess the quality of the 4 studies, we used a protocol designed by Kmet et al. (30). There were 14 criteria assessed with a score to be given between 0 and 2 (0 for a “no” answer, 1 for a partial answer, and 2 for a “yes” answer). Quality assessment was performed by three independent reviewers and then compared. Any disagreement was discussed with the entire team of authors. As shown on **Table 2**, the scores reported were summed for the 4 articles. These scores are intended to be indicative, as the assessment had no impact on the selection process.

## Results

### Characteristics of the studies and content of interventions

The four studies included are summarized in Table 1. Participants were aged between 8 and 28 years. In one study, adolescents up to 18 were part of a target group which included pediatric patients as young as 8 (33). By contrast, two studies included young adults up to 21 (32) or 28 (34). In only one selected study, the age limits were restricted to the adolescent period, e.g., between 10 and 14 (31).

**Table 1:** Summary of the included studies.

**Authors**	**Country**	**Control group**	**Design**	**Follow-up**	***N***	**Age**	**Condition**	**Name of intervention**	**Intervention duration**	**Dependent variables**
Creedy et al. (31)	Australia	No	Pre-post design	No	12 children-parent dyads	10–14 (*M* = 11.2)	Diabetes Friedriechs ataxia Cystic fibrosis Visual impairment Caeliacs disease Lymphedema Asthma	Child and Parent Support Program (CAPS)	8 weeks	Family social climate Parental perceptions of child's condition Self-esteem Anxiety Depression Coping strategies
Xenakis et al. (32)	USA	No	No pre-post design	No	28 young women	14–21 (*M* = 17.4)	Cerebral palsy Spina bifida Partial leg disability Metatropic dysplasia Mitochondrial disease Multiple sclerosis Muscular dystrophy Osteogenesis imperfecta Progressive myclonus epilepsy Spinal muscular atrophy Traumatic brain injury	Young women's Program	12 weeks	Program evaluation Personal health and wellness plans
Last et al. (33)	The Netherlands	No	Pre-post design	At 6–8 months	109 pediatric patients, of which 59 adolescents aged 12–18	8–18 (*M* = 13.4)	Inflammatory Bowel Disease Cancer Diabetes Others	Op Koers Op Weg for adolescents	6 weeks	Interventions goals Disease related skills Use of relaxation Social competences Positive thinking Coping Behavioral and emotional problems Quality of daily functioning Anxiety Self-worth
Van der Stege et al. (34)	The Netherlands	No	No pre and post design	No	85 adolescents	11–28 (*M* = 16.7)	Physical disabilities HIV Neuromuscular diseases	SeCZ TaLK	The time of the game	General attitudes Importance of discussing sexuality and relationships Feeling free to express Opinions during the game Appreciation of the game Feasibility of the game

The number of chronic conditions represented in the samples of participants included in each study ranged from 3 (34) to 11 (32) different conditions. All participants were outpatients. The durations of the interventions varied from the time taken to play a game (34) to up to 12 weeks (32).

Congruently with our inclusion criteria, the interventions described in the selected articles did not take into consideration the nature of the condition, nor did they target the condition directly or indirectly. In order to promote the health of chronically ill adolescents beyond specific illness and self-management education, the specific areas of intervention that had been defined by the authors were the empowerment of adolescents by developing functional coping strategies (31, 33), the development of general knowledge about health and wellness (32), and the communication about sexuality and intimate relationships to develop of a healthy attitude toward sex (34).

The study of Creedy et al. (31) aimed at developing coping skills through peer-support. They included parents in the intervention, in parallel sessions. The three other interventions were addressed to adolescents and young adults (32–34). Although in one of these studies (33), parents were also invited to participate in separate workshops, there are no details about their participation. The *Young women's program* (32) and the *SeCZ TaLK board game* (34) are psycho-educational programs, which provide information and teach participants general knowledge, respectively about health and sexuality. The intervention used by Last et al. (33) focused primarily on developing the active use of functional coping strategies.

### Evaluation of the targeted outcomes and quality assessment

The four included studies provided diverse information on the intervention. Whereas two studies provided content details and concrete examples (33, 34), in two other studies, the general themes of the sessions or the workshops were listed with little concrete details about the content (31, 32). Although participation is a key concept in health promotion, only in one of the selected studies was the prototype of the intervention developed with the participation of the adolescents who were going to benefit from it (34).

The quality assessment of the studies is reported in Table 2. The results show that the aims and the study design of the four included studies were easily identified and the design seemed to be appropriate to address the research questions. Among the four included studies, none were randomized and there were no control groups. Two studies had a pre-post design (31, 33) and one of these had a follow-up (33). Assessments contained intervention satisfaction/evaluation in the two studies with no pre-post design (32, 34). In the two other studies, authors investigated family issues, such as the family social climate (31), and diverse aspects of psychological functioning, such as coping strategies, self-worth, children's emotional problems (31, 33). The authors assessed their intervention, using either their own customized questionnaires alone (32, 34), or their own questionnaires in combination with standard validated questionnaires (31, 33). Little information was found about the validity and the reliability of the customized questionnaires, except for the one used by Last et al. (33).

**Table 2:** Description of the Quality Assessment according to Kmet et al.'s quality criteria (28).

		**Yes**	**Partial**	**No**	**Not applicable**
1	Question/objective sufficiently described?	4	0	0	0
2	Study design evident and appropriate?	4	0	0	0
3	Method of subject/comparison group selection or source of information/input variables described and appropriate?	0	4	0	0
4	Subject (and comparison group if applicable) characteristics sufficiently described?	3	1	0	0
5	If interventional and random allocation was possible, was it described?	0	0	0	4
6	If interventional and blinding of investigators was possible, was it reported?	0	0	0	4
7	If interventional and blinding of subjects was possible, was it reported?	0	0	0	4
8	Outcome and (if applicable) exposure measure(s) well defined and robust to measurement/misclassification bias? Means of assessment reported?	2	1	1	0
9	Sample size appropriate?	2	2	0	0
10	Analytic methods described/ justified and appropriate?	2	2	0	0
11	Some estimate of variance is reported for the main results?	2	1	1	0
12	Controlled for confounding?	0	2	0	2
13	Results reported in sufficient detail?	2	2	0	0
14	Conclusions supported by the results?	4	0	0	0

## Discussion

The present paper aimed to review any non-disease-specific hospital-based intervention designed to promote general health in chronically ill teenagers. However, our literature search revealed a surprisingly small number of relevant studies. Like Sawyer et al. (27), in their inspiring work about the challenges that health services and health professionals face as they care for adolescent patients, we found that non-disease specific studies focusing only on adolescents (as opposed to pediatric groups or adult groups comprising a sub-group of adolescents) are still lacking. In contrast to the growing evidence regarding the general health needs of adolescents living with chronic conditions (27, 35), health promotion for these adolescents remains an under-investigated area of research, and probably even of practice.

As mentioned in the Methods section, our initial search had been limited to the 2003–2014 period. However, while in the process of preparing our manuscript, we conducted a new systematic search over the 2014–2017 period. As already mentioned, this search did not yield any other results as all articles that were found to be potentially relevant to the health promotion objective for chronically ill adolescents in clinical settings were excluded on the basis that they were disease-specific, i.e., targeting specific groups of adolescents that shared the same chronic condition. We did however find one study about a non-specific intervention, published by van der Stege et al. (36). Another study by the same authors and regarding the same intervention (34) is among the 4 articles selected for our review. Both studies report the use of a board game (the SeCZ TaLK) to facilitate communication around sexual health issues for different groups of adolescents in different settings (34, 36). Yet, the 2016 study was excluded because its aim was not to evaluate the effectiveness of the intervention for the young people, but the experience of the professionals who had used the game, in terms of acceptability and feasibility, which is outside the scope of our review. Interestingly however, the results of the 2016 study show that the health promotion intervention based on the use of the game was more readily implemented by professionals working in contexts other than hospitals, such as professionals working in special schools or rehabilitation centers. The authors point to contextual and organizational factors that fail to create opportunities to use the game in hospitals, such as a lack of training on the part of the health care providers, who are not prepared enough to work together with groups of patients, and to communicate about intimate issues in relations to their young patients' general health (37).

Another factor explaining the paucity of studies that was found to be relevant to our research objective could be that a great number of interventions initialized in hospitals are tailored to the specific challenges of each adolescent and their family. If so, it is likely that interventions promoting general health in adolescents with a chronic condition exist in clinical practice but are rarely the subject of publications. Therefore, encouraging the publication of single-case studies would be worthwhile (38). Indeed, there is a growing acknowledgment that single-case studies are a fundamental component of evidence-based practice in psychology (37, 39). A systematic and well-documented single-case formulation offers fruitful information (40), such as specific reactions of participants to a part of the intervention. Such information could translate into recommendations for the development, implementation and adaptation of group interventions, with the purpose to promote better adolescent health in clinical settings.

Moreover, it could be that most interventions that are initiated in hospitals and that target primary prevention, health education or health promotion are not labeled as such. Indeed, health promotion goals, to some extent, are probably pursued as part of patient education interventions (i.e., self-management support or adherence enhancing interventions) on the one hand, and psychological interventions on the other. Although such interventions are primarily directed at tertiary prevention (i.e., alleviating the burden and complications associated with an existing condition) and were therefore excluded from our selection, they may encompass specific primary prevention goals, and thus overlap with broader health education and health promotion interventions. Thus, looking at our selection of studies with a health psychology lens, these could be also labeled as either (1) emotional support, (2) psycho-educational program, or (3) skills-based programs, in concordance with the typologies proposed by Plante et al. (41) and Sansom-Daly et al. (42). The findings from a systematic literature review on psychological interventions for adolescents and young adults living with a chronic illness have shown these to have a positive impact (42). To illustrate, mindfulness-based interventions (MBI) are evidenced-based approaches that aim at improving the general wellbeing of adolescents (43). Some studies about MBI in medical settings for adolescents begin to emerge in the literature (44).

### Strengths, limits and perspectives

The main strengths of this review are the methodology employed and the rigorous approach to the selection of the included studies. Multiple authors were independently involved in the selection process, data extraction and quality assessment. However, as indicated in the Results section, the methods employed to assess the interventions described in our selection of articles are not scientifically robust enough to allow for a generalization of the findings in terms of effectiveness. Despite these methodological shortcomings and the paucity of studies that actually met our inclusion criteria, what has been demonstrated is the clinical relevance and feasibility of developing non-disease specific health promotion interventions in clinical settings. Moreover, congruently with health promotion principles and values, the interventions described in our selection of articles, targeted positive health determinants, in terms of personal skills that contribute to psychosocial resiliency, rather than aiming to change behaviors (45). Unfortunately, the lack of relevant reported details did not allow us to highlight the key factors and mechanisms associated with successful interventions for health promotion, targeted at chronically ill adolescents attending health care settings. The transferability of interventions that have proven to be effective in settings other than medical settings, for instance school-based or community based interventions, is beyond the scope of our discussion. However, reviewing the evidence of health promotion and primary prevention interventions that target healthy adolescents, and that are built on traditional health education principles and strategies such as participative approaches, support groups and peer education, attention to protective factors and not only risk factors, would help explain what may work, under which circumstances, in hospitals as well. In fact, there is some empirical evidence that peer support groups outside the hospital are effective in supporting chronically ill adolescents cope with the psychosocial challenges inherent to growing up with a chronic condition (46). As most chronically ill adolescents are not in the hospital, innovative ways of engaging youth in health promotion oustside the hospital, including interventions using social media, need to be developed to reach out to the large community of chronically ill adolescents (47).

## Author contributions

IA has designed the study, selected and read the articles, drafted the introduction, methods and discussion sections, finalized and approved the final manuscript; SD has selected and read the articles, analyzed the extracted data, drafted the results section, and approved the final manuscript; ML has selected and read the articles, analyzed the extracted data, finalized the results section, drafted the discussion section, and approved the final manuscript.

### Conflict of interest statement

The authors declare that the research was conducted in the absence of any commercial or financial relationships that could be construed as a potential conflict of interest.
